# Description of a new species of *Icosta* Speiser , 1905 (Diptera: Hippoboscidae) from Southern Vietnam with the updated key to the subgenus *Icosta*

**DOI:** 10.1016/j.ijppaw.2024.101026

**Published:** 2024-11-30

**Authors:** Aleksandra Yatsuk, Emilia Nartshuk, Andrey Bushuev, Anvar Kerimov, Nguyễn Văn Linh, Oleg Tolstenkov, Alexandr Matyukhin

**Affiliations:** a A.N. Severtsov Institute of Ecology and Evolution, Russian Academy of Sciences, 33 Leninskiy Av., 119071 Moscow, Russia; b Zoological Institute of the Russian Academy of Sciences, Universitetskaya emb. 1, 199034 Saint Petersburg, Russia; c Department of Vertebrate Zoology, Faculty of Biology, Lomonosov Moscow State University, Leninskie Gory 1/12, Moscow 119234, Russia; d Southern Branch of Joint Vietnam-Russia Tropical Science and Technology Research Center, 3 Street 3/2, 10 District, Ho Chi Minh City, Viet Nam; e Michael Sars Center, University of Bergen, Thormøhlensgt. 55, 5008 Bergen, Norway

**Keywords:** Diptera, Hippoboscidae, Louse flies, New species, *Icosta*, Vietnam

## Abstract

The family Hippoboscidae, commonly known as “louse flies,” comprises pupiparous Diptera that are ectoparasites of birds and mammals, with significant impacts on their hosts and epidemiological importance. The louse fly fauna of Vietnam is relatively understudied compared to other countries in the Southeast Asia region. In this study, we describe a new species of the genus *Icosta* Speiser, 1905 (Diptera: Hippoboscidae), *Icosta korzuni***sp. n.**, collected from the lesser coucal *Centropus bengalensis* (Gmelin, JF, 1788) in Cat Tien National Park, Vietnam. This new species is distinguished from other *Icosta* species by the morphology of laterite 3, wing length, arrangement of wing microtrichia, body coloration, and its geographical distribution. Additionally, we provide an updated key to the subgenus *Icosta* Speiser, 1905.

## Highlights

• Described a new species, *Icosta korzuni* sp. n., from Cat Tien National Park, Vietnam.
• Reported the first avian louse fly species described from Vietnam.
• Provided an updated identification key for the subgenus Icosta (Diptera: Hippoboscidae).

## Graphical abstract

**Figure:**
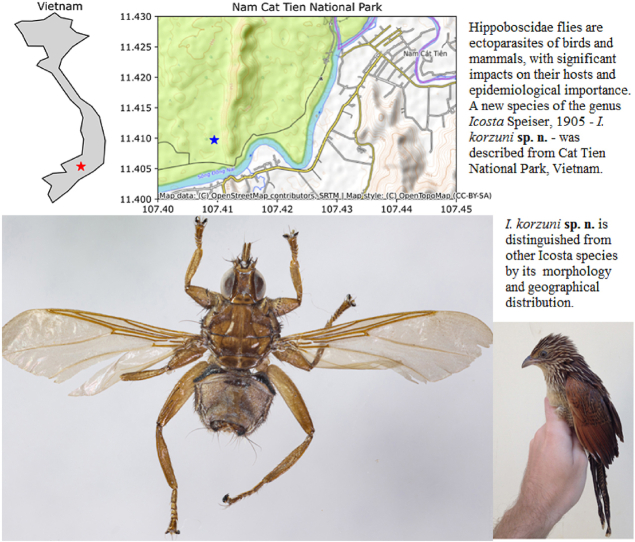

## 1 Introduction

Tropical rainforests are home to the planet's richest biodiversity (Mittermeier et al., 2003). However, these regions are increasingly threatened by deforestation and other environmental disturbances, leading to ongoing habitat loss that endangers numerous species, including many yet to be described and cataloged (Webb et al., 2010). Among tropical organisms, parasitic insects remain understudied, although recent efforts have led to the description of several new species (Madrid et al., 2020). Certain groups, like louse flies, have attracted even less attention despite their ecological significance.

The family Hippoboscidae, first described by Samouelle in 1819, includes over 213 species (Dick, 2006; Oboňa et al., 2022). These flies are obligate blood-feeding ectoparasites of mammals and birds (Hutson, 1984; Doszhanov, 2003) and serve as vectors for various diseases (Bequaert, 1955; Doszhanov, 1980; Gancz et al., 2004; Farajollahi et al., 2005; Čisovská Bazsalovicsová et al., 2023). They can act as specialized vectors for blood parasites such as *Haemoproteus* sp. and *Trypanosoma* sp. (Valkiunas, 2004; Fischer and Chakarov, 2024). In addition to their role as disease vectors, Hippoboscidae are known to transport phoretic mites (Hill et al., 1967; Philips and Fain, 1991) and feather lice (Lee et al., 2022).

One of the most prominent genera within the family, *Icosta* Speiser, 1905, is the largest genus in Hippoboscidae and comprises approximately 53–65 species (Maa, 1969c, 1969b; Dick, 2006). This genus is subdivided into five subgenera: *Ardmoeca* Maa, 1969, *Gypoeca* Maa, 1969, *Icosta* Speiser, 1905, *Ornithoponus* Aldrich, 1923, and *Rhyponotum* Maa, 1969a, Maa, 1969b, Maa, 1969c (Maa, 1969b; Dick, 2006). Species from the subgenus *Icosta* are found in the subtropical and tropical regions of Asia, Oceania, and Africa, with 23 species currently recognized (Maa, 1969c, 1969b; Dick, 2006). These species are distinguishable by several morphological features, including a largely bare mesonotum, bare ventral surfaces of the femora (except for a few marginal setae), and strongly pronounced frontal and vibrissal processes, which are separated by a distinct notch or incision (when processes features are not so, than tarsomeres 3 and 4 of foreleg are distinctly asymmetrical, or metasternal process is well developed, or frontal process in dorsal view is strongly narrowed apicad and with inner margin distinctly curved in S-shape). The palps are never very short (Maa, 1969c).

In Vietnam, only one species of mammal-associated louse fly has been described to date (Maa, 1969a), and relatively few species associated with bird hosts have been documented (Maa, 1969b, 1977). To date only *I. trita* Speiser, 1905 from the subgenus *Icosta* was collected in Vietnam (Maa, 1977). Considering the diversity of climatic zones, habitats, and bird species in this country, it is likely that additional, as yet undiscovered, species of louse flies exist.

The aim of this study is to describe a new species of *Icosta* collected from birds in Vietnam, representing the first description of bird-associated louse flies in this country. This work also seeks to contribute to a broader understanding of the biodiversity of Hippoboscidae in Vietnam.

## 2 Materials and methods

### 2.1 Study area

**Fig. 1:**
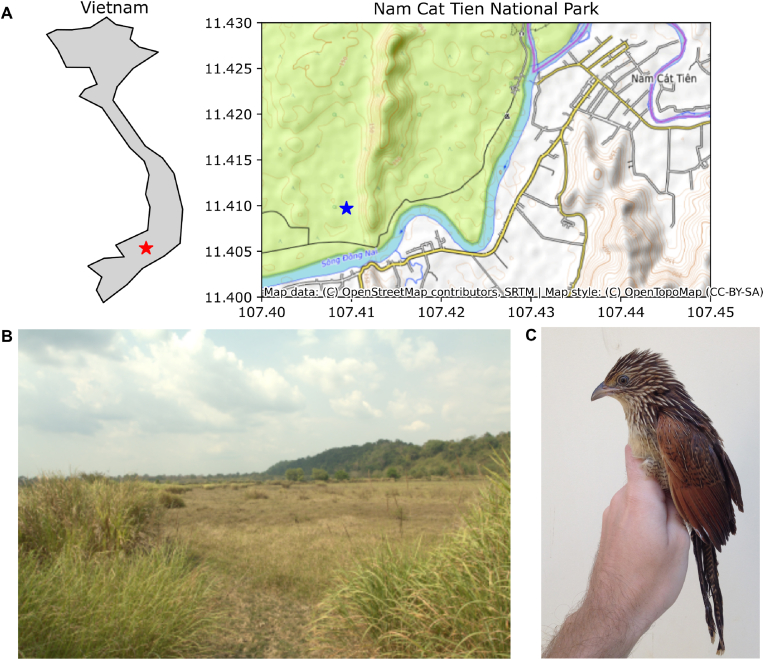
Collection location and the host. A. Position of Nam Cat Tien national park in Vietnam (red star) and the collection spot in the national park (blue star). B. Habitat where the host and the specimen were sampled. C. Host *Centropus bengalensis* (Gmelin, JF, 1788).

Study was performed in Vietnam in Cát Tiên National Park (11.420517; 107.425333), located to the north-east of Ho Chi Minh City in Dong Nai Province, Southern Vietnam (Fig. 1 A). The climate of the region is a tropical monsoon with an annual ambient temperature of about 26 °C (Deshcherevskaya et al., 2014; Kuricheva et al., 2021). Cát Tiên National Park has several land cover types: primary evergreen forest, primary and secondary mixed and deciduous forest, bamboo forest, agricultural land and seasonally flooded grassland (Vandekerkhove et al., 1993; Blanc et al., 2001; Kuznetsov and Kuznetsova, 2011). It is a part of the greater Dong Nai UNESCO Biosphere Reserve and is located in the South Vietnamese Lowlands Endemic Bird Area (EBA). Being a home for about 350 species of birds (including four endemics of Vietnam and 16 species from the IUCN Red List), it is a key area for avian biodiversity among the lowland forests of Southern Vietnam (Tuong and Van La, 2006). Cát Tiên is one of the largest national parks in the country and is included in the IUCN Green List of Protected and Conserved Areas.

### 2.2 Sampling

Birds were caught with mist nets (Bushuev et al., 2018, 2021) at the end of the dry season and the beginning of the rain season February–May 2018–2019. Juvenile birds and females with a strongly pronounced brood patch were released immediately after quick manual check for flies. Most of the birds were sampled for louse flies using fumigation chamber and ethyl acetate (Clayton and Drown, 2001).

During the entire study, 247 birds representing 64 species of over 10 avian families were captured, and 16 Hippoboscidae flies were collected by O. Tolstenkov, A. Bushuev, N. V. Linh, and A. Kerimov. Among these, one specimen of the newly described Icosta species was collected from *Centropus bengalensis* representing the only representative of the Icosta species in our study. Notably, two individuals of *C. bengalensis* were captured during the survey, one carrying the new Icosta species and the other carrying a female from the genus Ornithophila. The material of collected flies was fixed in 96% ethanol.

### 2.3 Ethic statement

The design of our study was approved by the ethics committee of Lomonosov Moscow State University [resolution of the committee N 84a]. We have exerted every effort to carry out our work in compliance with present international ethical standards. All our experiments were intravital and did not require prolonged treatment and handling of birds. None of the avian species from our study were included in “Threatened” category of the IUCN Red List of Threatened Species. Since louse flies, being parasites, are not a protected species, no ethical approval was required for their research.

### 2.4 Species identification

Species identification of the samples was carried out according to the key of Maa (1969c). The morphological study was conducted using optical microscope Keyence VHX-1000 (Japan) at the Joint Usage Center « Instrumental methods in ecology » at the Institute of Ecology and Evolution, Russian Academy of Sciences. Morphological terminology follows Maa (1969b) and Hutson (1984). For illustration purposes images were taken with Canon EOS 90D and Canon EOS M6 Mark II cameras with a Canon EF 100 mm/2L Macro lens, stitched and processed using Helicon Focus 7 software.

The key of Maa (1969c) has been modified by adding a new species and using the wing length for identification. More attention was paid to geographic distribution data, level of laterite 3 definable and arrangement of microtrichia on the wings. Head features were used less.

## 3 Results

**Description.**

Order **Diptera Linnaeus, 1758**

Family **Hippoboscidae Samouelle, 1819**

Subfamily **Ornithomyinae Bigot, 1853**

Genus ***Icosta*** Speiser, 1905

Subgenus *Icosta* Speiser, 1905

**Fig. 2:**
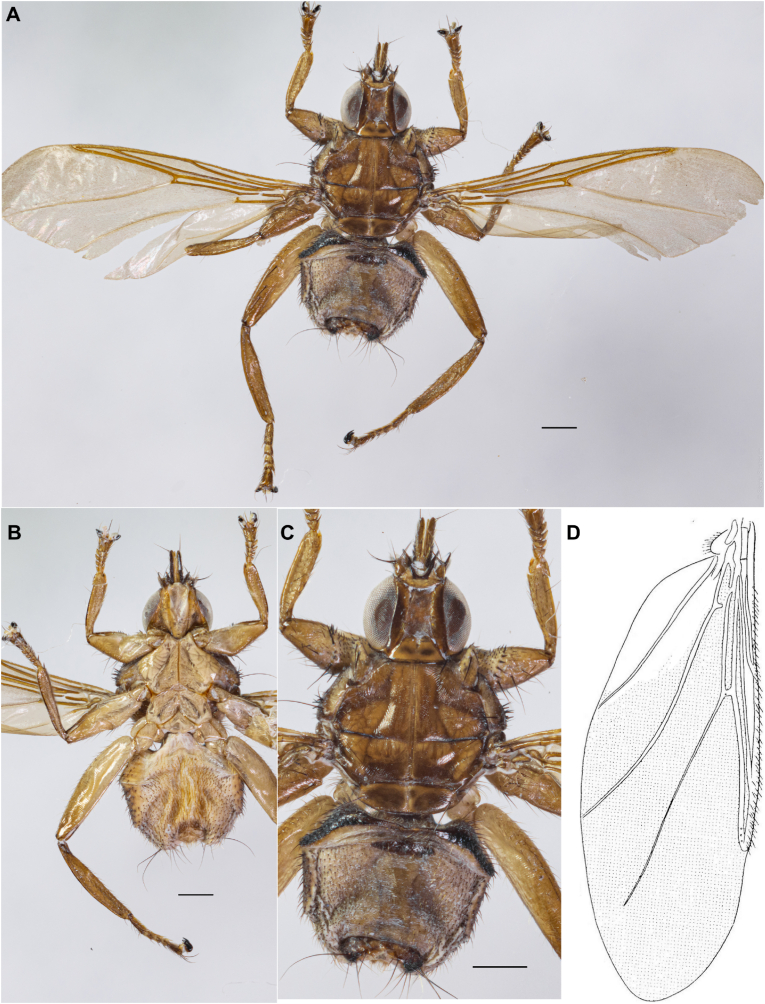
*Icosta korzuni***sp. n.** female (holotype): A. general view, dorsal side; B. ventral side; C. dorsal side; D. wing drawing. This new species is distinguished from other *Icosta* species by the morphology of laterite 3, wing length, arrangement of wing microtrichia, body coloration, and its geographical distribution. Scale bars: 0.5 mm.

**Fig. 3:**
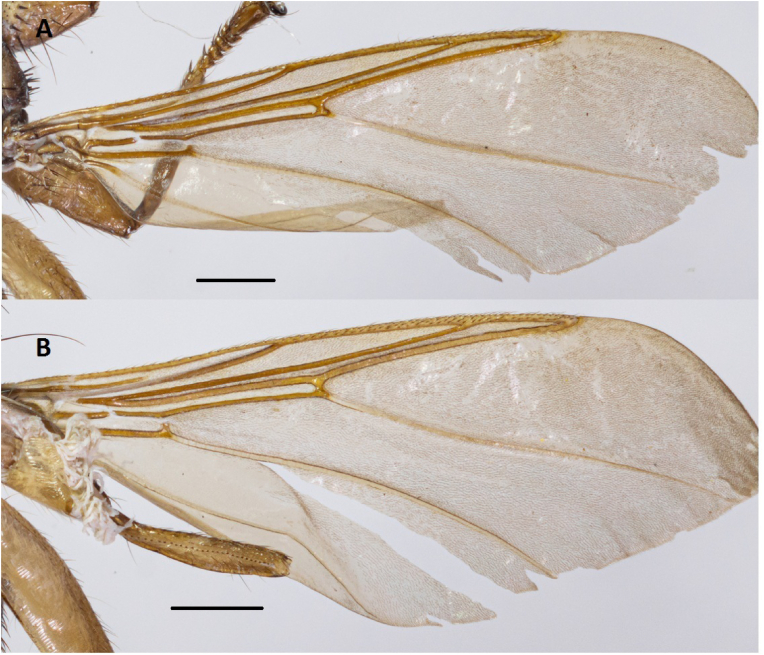
Wings of *Icosta korzuni***sp. n.** female (holotype): A. right wing; B. left wing. Scale bars: 0.5 mm.

*Icosta korzuni* Yatsuk, Matyukhin et Nartshuk **sp. n.**

(Fig. 2, Fig. 3 A, B)

**Type material examined:** Holotype, 1 female: Vietnam, Nam Cát Tiên National Park (11.420517; 107.425333), collected from the lesser coucal *Centropus bengalensis* (Gmelin, JF, 1788), caught in the grasslands on April 16, 2018 (Fig. 1 C) (O.O. Tolstenkov).

The holotype in ethanol is deposited in the collection of the Zoological Institute of the Russian Academy of Sciences, St. Petersburg (inventory number INS_DIP_0001110).

**Description.** Head and thorax length combined 2.5–2.6 mm (without palps). Palp length 0.2 mm.

Head with posterior part located between humeral tubercles and slightly covering anterior margin of thorax. Eye one-third as wide as head. Vertex on posterior margin is approximately 2 times wider than eye. Parafrontals wide. The setae on the parafrontals are yellow. Ocelli are absent. Width of inner orbit almost equal to one-half of mediovertex width. Posterior margin of lunula rounded. Lunula horns located between antennae, clearly separated from lunula. Horns form U-curve. Palp length two times more than second antennal segment. Antennae brown. Ventral side of head light with dark triangles.

Mesonotum light brown. Humeral tubercles approximately cone-shaped, protruding anterolaterally. Prescutum densely feebly striate all over. Longitudinal (medial), transversal and scuto-scutellar sutures clearly visible. Transversal suture interrupted in middle; longitudinal suture almost reaching scuto-scutellar suture. Scutellum posterolaterally rounded, posteriorly distinctly convex. Interdistance of bases of scutellar setae almost 3 median length of scutellum. Setae of mesonotum: row of short light laterocentral setae, 1 long light brown notopleural seta. Setae of scutellum: thin light setae forming fringe on its posterior margin; 1 long black seta at each edge of scutellum. Ventral side of thorax light.

Wing length 4.8–5.0 mm. Wing with 2 transverse veins and correspondingly with 2 closed basal cells 1bc and 2bc. Longitudinal veins R1, R2+3 and R4+5 connecting with Costa at acute angle. Section on Costa between juncture of R1 and R2+3 twice as long as section between juncture of R2+3 and R4+5. The length of cell 2bc is approximately 2 times shorter than cell lbc. Microtrichia covering cells 3r, 1m, part of 2m+1a and short piece of 2a. In 2m+1a microtrichia cover approximately half of cell, being absent in basal part and along vein 2A. In cell 2a microtrichia occupy small triangle at the top of vein 2A.

Legs light. Femora strong. Venter of all femora bare, excepting marginal setae. Claws bifid. Empodium and paired pulvilli not reduced.

Abdomen covered with short setae. Tergite 1 + 2 with straight posterior margin. Tergite 3 approximately one third as wide as abdomen. Tergites 4, 5 absent. Tergite 6 strongly constricted at middle, its posterior margin concave at middle. Tergite 6 with 4 strong black setae at each half. Laterite 3 dark and highly visible. Setae on venter side of laterite 3 similar to those on adjacent membranous area.

**Comparison***.* The new species belongs to the subgenus *Icosta*. *I. acromialis* (Speiser, 1904) and *I. fenestella* Maa, 1969a, Maa, 1969b, Maa, 1969c are the closest Asian species in morphological features to *I. korzuni* **sp. n.** *I. acromialis* differs from the new species in arrangement of wing microtrichia, that completely cover the cells 1m and 2m+1a, in prescutum strongly striated all over and in dark body color. *I. fenestella* differs from the new species in arrangement of wing microtrichia, that in the cell 2m+1a do not reach vein 2A and form a small bare stripe in cell 1m and morphology of laterite 3, that is hardly definable. Additionally, it differs in paler body color and length of cell 2bc, that is clearly less than 1/2 lbc (Maa, 1969c). Distinguishing features for *I. Icosta* species in comparison to *I. korzuni* **sp. n.** are given in Table 1. Other *Icosta* species from this subgenus noticeably differ from the new one particularly in the morphology of laterite 3, wing length, arrangement of wing microtrichia and geographic distribution.

**Table 1:** Distinguishing features, based on Maa (1969c), for *I. Icosta* species in comparison to *I. korzuni***sp. n.**

Species	Arrangement of wing microtrichia	Morphology of laterite 3	Wing length (mm)	Body color	Geographical distribution	Tergite 3 morphology
*I. chalcolampra* (Speiser, 1904)	are absent in a triangular area of the basal part of the cell 1m	poorly sclerotized	5.1–5.7	dark	a	a
*I. corvina* Maa, 1969	bare on posterior 1/3 of 2m+1a and entirety of 2a cells and apical margin of cell 3r	a	5.5–5.9	dark	a	a
*I. dioxyrphina* (Speiser, 1904)	broad bare stripe on apical margin of cells 3r, Im and 2m + la	a	5.8–6.9	very dark	Papuan Subregion	a
*I. jactatrix* Maa, 1969	cell 2m+1a with only very small triangular microtrichiate patch at antero-apical corner	a	a	a	New Guinea	Small size
*I. longipalpis* (Macquart, 1835)	covering all wing cells excepting 2a	a	8.5–9.5	dark	a	a
*I. spinosa* Maa, 1969	covering all cells except posterior 1/3 of 2m+1a and entirety of 2a, with narrow bare stripe on apical wing margin	a	6.1–6.4	a	a	membrane between tergite 3 and spiracles 3 with 3–4 strong setae
*I. tarsata* Maa, 1969	a	not definable	5.7–5.9	fairly dark	a	a
*I. wenzeli* Maa, 1969	covering all cells except 2m +1a (posterior 1/2) and 2a, 1m with short narrow bare stripe near base	poorly sclerotized, some setae on laterite 3 are more strong than adjacent	a	a	a	a

a Similar to *I. korzuni***sp. n**. or unknown.

**Hosts**. The new species was collected from lesser coucal *Centropus bengalensis* (Gmelin, JF, 1788).

**Etymology.** The new species is named in honor of the first general director of the Joint 10.13039/100020215Russian-Vietnamese Tropical Research and Technological Center, Leonid Petrovich Korzun, who actively supported research in tropical ecology and biodiversity.

The key by Маа (1969c) to the species of the subgenus *Icosta* can be updated as follows:

1. Fly from Africa … … … … … … … … … … … … … … … … … … … … … … … … … …. … … … …. 2
  – Fly from Asia … … … … … … … … … … … … … … … … … … … … … … … … … … … … … . 7
  – Fly from Pacific Ocean Islands of Papuan Subregion, Australia, New Zealand … . 19
2. Vein R2+3 normal, evenly or nearly evenly narrow, its combined width with C, after merging together, not or hardly wider than apical section of R4+5 … … … … … … … … … … 3
  – Vein R2+3 abnormal, its apical 1/2 suddenly widened and coalescent with C, their combined width, after coalescence, nearly 2 times as wide as apical section of R4+5. Wing with fairly broad bare stripe along anal margin … . *I. coalescens* (Maa, 1964)
3. Cell 2m+1a uniformly setulose, at most with very small bare area near base. 2a with tiny patch of setulae at its anteroapical corner. 2bc only 1/3 as long as 1bc. Wing 4.8 mm long … … … …. 4
  – Cell 2m+1a bare at posterior 1/3. 2a entirely bare. 2bc is 1/2 to 2/5 as long as 1bc … ….................................................................................................................. … …. 5
4. Lateral areas of abdominal syntergite 1 + 2 are not highlighted. Posterior margin of tergite 1 + 2 with only pale soft setae. Tergite 3 poorly sclerotized. Laterite 3 not definable … … … …. … … … … … … … … … … … … … … … … … … … … … … ….… … … …. *I. subdentata* Maa, 1969a, Maa, 1969b, Maa, 1969c
  – Lateral areas of abdominal syntergite 1 + 2 dark brown. Posterior margin of tergite 1 + 2 with series of black stiff setae near its lateral ends. Tergite 3 well sclerotized. Laterite 3 weakly definable … … … … … … … … … … … … … … *I. humilis* Maa, 1969a, Maa, 1969b, Maa, 1969c
5. Frontal process unusually long … … … … … … … … … … … … … … … … … … … … … ….… … 6
  – Frontal process short … … … … … … … … … … … … … . *I. recessa* (Maa, 1964)
6. Microtrichia do not cover a triangular area of the basal part of the cell 1m. In cell 2m+1a microtrichia cover only apical angle. Wing 5.5 mm long … … … *I. malagasii* Maa, 1969a, Maa, 1969b, Maa, 1969c
  – Wing microtrichia are arranged differently … … … . *I. mecorrhina* (Maa, 1964)
7. Wing length 6 mm or more … … … … … … … … … … … … … … … … … … … …...… … … … …. 8
  – Wing length less than 6 mm … … … … … … … … … … … … … … … … … … … … … … . 12
8. Wing length 8.5–9.5 mm. Color of body dark, wing fuscous*.* Palpus is 5/7 times as long as distance between frontal notch and occipital margin ….. *I. longipalpis* (Macquart, 1835)
  – Wing length less than 8 mm … … … … … … … … … … … … …... … … … … … … … … … 9
9. Microtrichia do not cover a triangular area of the basal part of the cell 1m. Wing 6.2–6.6 mm long … … … … … … … … … … … … … … … … … … … … … ….… …. *I. elbeli* Maa, 1969c, Maa, 1969b, Maa, 1969a
  – Microtrichia cover the basal part of the cell 1m almost completely or there is no triangular bald area … … … … … … … … … … … … … … …...… … … … … … … … … … … 10
10. Palpus 1/2–1/3 longer than frontal process … … … … … ….… … … … … … … … … … … …. 11
  – Palpus not longer, or hardly so than frontal process and much shorter than mediovertex. Wing 6.1–6.4 mm long … … … … … … … … … . *I. spinosa* Maa, 1969a, Maa, 1969b, Maa, 1969c
11. Microtrichia do not cover at least a quarter of the apical part of the cell 1m. Wing 6.2–6.6 mm long … … … … … … … … … … … … … … … … … ….… … … … … …. *I. bucerotina* Maa, 1969a, Maa, 1969b, Maa, 1969c
  – Microtrichia cover the cell 1m almost completely. Wing 6 mm long …..................................................................................... *I. bicorna* (Ferris, 1927)
12. Microtrichia completely cover the cells 1m and 2m+1a. Wing 4.6–5.2 mm long … … … ……. … … … … … … … … … … … … … … … … … … … … *I. acromialis tuberculata* (Ferris, 1927)
  – There are some bare areas in the cell 2m+1a … … … … … … … … … … … … … … … … 13
13. Laterite 3 dark and highly visible. Wing length 4.8–5.0 mm … … ….… … … *I. korzuni* **sp. n.**
  – Laterite 3 is less noticeable or wing length is smaller with fairly dark body color … ….................................................................................................................... …. …. 14
14. Wing length 5 mm or more … … … … … … … … … … … … … … … … … … … …....... … … … … … 17
  – Wing length less than 5 mm long … … … … … … … … … … … … … … …...… … ….… 15
15. Frontal process in profile broadly rounded, not projecting, not clearly separated from vibrissal area except in color and in degree of sclerotization. Wing 4.2–4.7 mm long … … … … … … … … … … … … … … … … … … … … … … … … … … … … … …. …. *I. wenzeli* Maa, 1969c, Maa, 1969b, Maa, 1969a
  – Frontal process in profile sharply projecting, toothlike, clearly separated from vibrissal area by deep sharp notch … … … … … … … … … … … … … … … … … … … … … 16
16. Setulose area of cell 2m+1a triangular, confined to antero-apical corner of that cell. Face and thoracic dorsum in fully matured specimens black with strong metallic lustre. Wing 3.5–4.2 mm long … … … … … … … … … … … … … … … … … …. … …. *I. trita* (Speiser, 1905)
  – Setulose area of cell 2m-+1a long, broad, extending along full length of apical abscissa of vein M3+4. Face and thoracic dorsum in fully matured specimens yellowish or reddish brown, lacking metallic lustre. Wing 4.2–4.8 mm long … … … … …. ….…....................................................................... … …. *I. fenestella* Maa, 1969a, Maa, 1969b, Maa, 1969c
17. Microtrichia almost completely cover the cell 2m+1a, with the exception of a bare strip along the vein 2A. Wing 5.5–5.9 mm long … … … … … … … … … … … …. *I. corvina* Maa, 1969a, Maa, 1969b, Maa, 1969c
  – There is much more bare area in the cell 2m+1a.… … … … … … … … …...… … … … 18
18. Basal 1/2 of anterior (inner) surface of femur 1 largely bare, with only few rows of setae along transmedian line and lower margin. Tarsus 1 weakly asymmetrical. Wing 5.7–5.9 mm long … … … … … … … … … … … … … … … … … … … … … … … … …. *I. tarsata* Maa, 1969a, Maa, 1969b, Maa, 1969c
  – Basal 1/2 of anterior (inner) surface of femur 1 fairly evenly setose. Tarsus 1 strongly asymmetrical. Wing 5.1–5.7 mm long … … … … … … …............................................... … … …. *I. chalcolampra* (Speise r, 1904)
19. Microtrichia completely cover the cell 2m+1a. Wing 4.6–5.2 mm long … … ….… … … … …. … … … … … … … … … … … … … … … … … … … … … …. *I. acromialis acromialis* (Speiser, 1904)
  –– There are some bare areas in the cell 2m+1a … … … … … … … … … … … … … ….… … 20
20. Scutellum normal, with evenly curved posterior margin … … … … … … … … … …...… … … 21
  –– Scutellum unusually short, posteriorly subtruncate. Wing 4.6–5.1 mm long … … … … … … … … … … … … … … … … … … … … … … … … … … …... *I. jactatrix* Maa, 1969a, Maa, 1969b, Maa, 1969c
21. Wing lacking bare stripe along anal margin … … … … … … … … … … … … … … … … … …. 22
  –– Cell 3r (in part), Im and 2m-+1a with fairly broad bare stripe along anal margin of wing. Wing 5.8–6.9 mm long … … … … … … … …. *I. dioxyrhina* (Speiser, 1904)
22. Setulose area of cell 2m+1a confined to apical corners or apical margin of that cell. Cell 1m either extensively bare at basal 1/2 or with 2–3 setulose stripes at apical part … … … …... . 23
  –– Setulose area of cell 2m+1a stretching along full length of abscissa 2 of vein M3+4. Cell 1m at most with tiny bare area or stripe at base … … … … … … … … … … . 24
23. Setulose area of cell Im divided into 2–3 stripes. Wing 5.2 mm long …. … *I. diluta* Maa, 1969a, Maa, 1969b, Maa, 1969c
  – Setulose area of cell Im undivide … … … … … …. *I. samoana* (Ferris, 1927)
24. Basal 1/2 of anterior (inner) surface of femur 1 largely bare, with only few rows of setae along transmedian line and lower margin. Tarsus 1 weakly asymmetrical. Wing 4.0–4.8 mm long … … … … … … … … … … … … … … … … … … …... …. *I. plana* (Walker, 1861)
  – Basal 1/2 of anterior (inner) surface of femur 1 fairly evenly setulose. Tarsus 1 strongly asymmetrical. Wing 5.1–5.7 mm long … *I. chalcolampra* (Speiser, 1904)

Key features of the subgenus *Icosta* are summarized in Table 1.

## 4 Discussion

To date, only one species of louse fly has been described from Vietnam (Maa, 1969b). Given the high biodiversity of birds and mammals in the country, as well as the unique habitats within Cát Tiên National Park (Craik et al., 2018; Masseloux et al., 2022), it is likely that many more species remain undiscovered. Despite our fieldwork's limited duration, we could identify a previously unknown species, *Icosta korzuni* **sp. n.** This finding underscores the richness of Vietnam's biodiversity and highlights the importance of continued exploration and documentation, particularly in under-studied regions like Cát Tiên. Such efforts are essential for building a comprehensive understanding of Vietnam's faunal diversity, especially in light of ongoing habitat changes and increasing pressures on tropical ecosystems.

Tropical Asia is the center of species diversity for the genus *Icosta*. The fauna of the louse flies in neighboring countries has been studied much more thoroughly (Maa, 1977), which makes new data on the louse flies biodiversity of Vietnam particularly valuable. Many migratory birds spend the winter in Vietnam or nearby regions (Tuong and Van La, 2006), making it critical to better understand the louse flies to detect the potential expansion of foreign species (Oboňa et al., 2022). To achieve this, robust data on the biodiversity of Hippoboscidae is essential. It would also be beneficial to review existing collections and, with the increased opportunities for gathering new material, revise the louse flies biodiversity in Oriental region.

In addition, louse flies are not only important components of biodiversity but also are vectors of wildlife diseases, including protozoan parasites *Haemoproteus* and *Trypanosoma*, viral and bacterial agents such as West Nile virus, and *Babesia* Starcovic 1893 (Farajollahi et al., 2005; Čisovská Bazsalovicsová et al., 2023), which can significantly impact host populations. Understanding the diversity and distribution of these vectors in tropical regions (Rahola et al., 2011) is crucial for assessing their epidemiological risks and potential effects on wildlife.

Future research should focus on expanding faunistic surveys, identifying vector-host associations, and evaluating their roles in disease dynamics to provide a deeper understanding of their ecological and epidemiological importance.

## CRediT authorship contribution statement

**Aleksandra Yatsuk:** Writing – review & editing, Writing – original draft, Investigation. **Emilia Nartshuk:** Writing – review & editing, Writing – original draft, Supervision, Methodology, Investigation, Data curation. **Andrey Bushuev:** Writing – review & editing, Methodology. **Anvar Kerimov:** Writing – review & editing, Methodology. **Nguyễn Văn Linh:** Writing – review & editing, Methodology. **Oleg Tolstenkov:** Writing – review & editing, Methodology. **Alexandr Matyukhin:** Writing – review & editing, Writing – original draft, Methodology, Investigation, Data curation.

## Conflict of interest

The authors declare no conflict of interest.
